# Early estimates of awareness and uptake of over-the-counter naloxone

**DOI:** 10.1093/haschl/qxaf107

**Published:** 2025-05-26

**Authors:** Mireille Jacobson, David Powell

**Affiliations:** Leonard Davis School of Gerontology, University of Southern California, Los Angeles, CA 90089, United States; RAND, Arlington, VA 22202, United States

**Keywords:** opioid crisis, naloxone access, over-the-counter medications

## Introduction

Improving access to naloxone, an effective opioid overdose reversal medication, is a cornerstone of the public health response to the opioid crisis in the United States. Many states have tried to broaden naloxone access by authorizing pharmacies to dispense and sell naloxone without a patient-specific prescription.^1^ In 2023, the FDA took a further step by approving 2 over-the-counter (OTC) naloxone nasal spray products, allowing individuals to purchase naloxone outside of the pharmacy setting.^2^ Despite the promise of this policy, limited evidence exists on consumer knowledge and engagement with OTC naloxone,^3^ which is needed to assess its effectiveness and evaluate additional policy options. This survey study examined knowledge and uptake of OTC naloxone.

## Methods

We surveyed a national US sample of 1515 adults aged 18 and older from June 10 to June 17, 2024 using Respondi, an online platform often used in academic research due to its high-quality national panels.^4,5^ Quotas were enforced based on sex, race/ethnicity, age, and region to match national demographics (see Supplement S1). Participants were informed near the beginning of the survey that Narcan is a brand-name version of naloxone and that naloxone can potentially reverse an overdose involving opioids and save a person's life.^6^

Participants were asked, “Are you aware that Narcan is now available over-the-counter, meaning it can be purchased directly, without asking a pharmacist to provide it?” Response options were: (1) “Yes, I have seen it available over-the-counter in a store,” (2) “Yes, but I have not seen it available over-the-counter in a store,” or (3) “No, I was not aware.” Narcan was the only naloxone product widely available OTC at the time of survey.

Respondents were also asked if they had ever carried naloxone; those responding affirmatively then selected all places where they had obtained naloxone, including “from a pharmacist (not over-the-counter)” and “over-the-counter at a retail outlet.”

We calculated proportions and 95% CIs of participants reporting awareness of, seeing, and purchasing OTC naloxone. We reported these statistics for the sample overall and stratified by self-reported overdose risk or risk that someone the respondent knows might overdose. Among participants, 71 (4.7%) self-assessed that they were very likely to overdose from opioid use and 169 (11.2%) indicated that they knew someone very likely to overdose.^7^ RAND's Human Subjects Protection Committee and the University of Southern California's Institutional Review Board approved the study.

## Results

**Figure 1.**
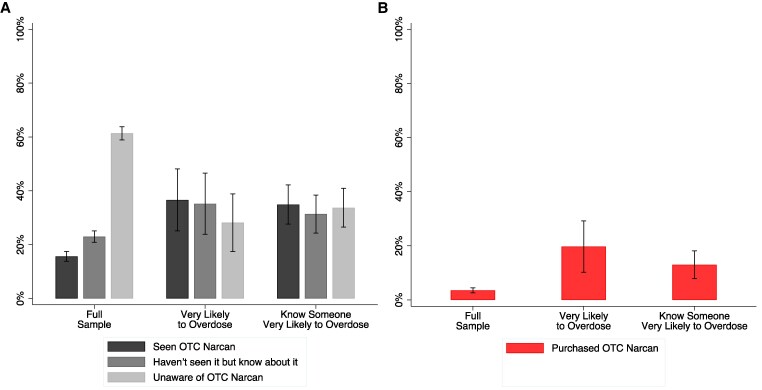
Percentage of respondents aware of over-the-counter Narcan and who have purchased it by overdose likelihood. Percentages and 95% CIs presented. A) Presents responses to “Are you aware that Narcan is now available over-the-counter, meaning it can be purchased directly, without asking a pharmacist to provide it?” Response options were: (1) “Yes, I have seen it available over-the-counter in a store,” (2) “Yes, but I have not seen it available over-the-counter in a store,” or (3) “No, I was not aware.” Results are presented for the full sample (*n* = 1515), those self-reporting that they are “very likely” to overdose from opioid use (*n* = 71), and those self-reporting that they know someone very likely to overdose from opioid use (*n* = 169). B) Respondents were first asked if they carry or have ever carried naloxone. Those answering affirmatively were then given a list of places in which they had obtained naloxone: (1) from a pharmacist (not over-the-counter); (2) from a clinic, hospital, or other medical setting; (3) at a harm reduction organization or public health department; (4) in the mail; (5) from a vending machine; (6) provided by a friend, family member, or someone else; (7) over-the-counter at a retail outlet; and (8) other. Respondents could select as many choices as applied. We report the number of respondents selecting “Over-the-counter at a retail outlet” as a share of the full sample (*n* = 1515), which includes those who have and who have not ever carried naloxone. We also report those selecting “Over-the-counter at a retail outlet” as a share of those self-reporting that they are “very likely” to overdose from opioid use (*n* = 71) and those self-reporting that they know someone “very likely” to overdose from opioid use (*n* = 169).

In the full sample, 15.6% (95% CI, 13.8%-17.5%) reported seeing Narcan available OTC, and an additional 23.0% (95% CI, 20.8%-25.1%) reported knowing that Narcan could be purchased OTC but had not observed it for sale (Figure 1). Among individuals who considered themselves very likely to overdose, 36.6% (95% CI, 25.1%-48.1%) reported seeing OTC Narcan and 35.2% (95% CI, 23.8%-46.6%) were aware of OTC availability without having observed it. Among those who reported knowing someone very likely to overdose, 34.9% (95% CI, 27.7%-42.2%) had seen OTC Narcan and 31.4% (95% CI, 24.3%-38.4%) were aware of OTC availability without having observed it.

Overall, 3.6% (95% CI, 2.6%-4.5%) reported obtaining naloxone OTC. Among those who perceived themselves as very likely to overdose from opioid use, 19.7% (95% CI, 10.2%-29.2%) had obtained naloxone OTC. Among those who know someone very likely to overdose from opioid use, 13.0% (95% CI, 7.8%-18.1%) reported obtaining OTC naloxone.

## Discussion

The introduction of OTC naloxone represents a potentially critical innovation to reduce opioid overdose deaths; yet, awareness and purchasing remain low in the general population. Overall awareness was relatively low: more than 60% of respondents were unaware that naloxone is available for purchase OTC. Importantly, however, individuals at higher perceived overdose risk, or who reported knowing someone at high risk, demonstrate greater awareness and higher purchase rates. Almost 20% of people who consider themselves very likely to overdose have purchased naloxone OTC and more than 70% knew about it or had seen it in stores.

The awareness findings underscore gaps in education about the availability of OTC naloxone. Public health campaigns and pharmacy-based initiatives could increase visibility. Low purchase rates may reflect barriers due to cost or stigma^8^ or due to access to low-cost alternative sources of naloxone.^9^ Policy interventions—such as insurance mandates or subsidies—may improve access and encourage wider adoption.

Future research should monitor trends in OTC naloxone awareness and purchases, assess barriers, and evaluate the impacts of targeted interventions. Limitations of this study include reliance on an online panel and self-reported behavior. Despite these limitations, our findings provide early insights into the initial public response to OTC naloxone.

## Supplementary Material

qxaf107_Supplementary_Data
