# Supplementary material for: Early estimates of awareness and uptake of over-the-counter naloxone
Source: Health Aff Sch. 2025 May 26;3(6):qxaf107. doi: 10.1093/haschl/qxaf107 (PMC12202286; doi:10.1093/haschl/qxaf107)
Supplement: qxaf107_Supplementary_Data [file qxaf107_supplementary_data.zip › supplement_OTC revision v1.docx]

**Supplement S.1**

**Section 1.1: Respondi Panel**

We employed an internet panel from Respondi, a well-established survey research firm, to recruit a cross-sectional sample of adults aged 18 and over. Respondi panels, which are maintained through active recruitment and validated authentication, have been used in many published studies.^1-9^ Panel members receive small incentives for survey participation. For the national sample, we set recruitment quotas by sex (male/female), race (White/non-White), age-group (aged 18-29, 30-39, 40-49, 50-59, 60 and over), and region (Northeast, Midwest, South, West) to achieve national representation on these important demographics. Respondents not in the US were dismissed from the survey and excluded from the analysis. We excluded respondents who failed an attention check (n=746), a commonly used approach to screen out individuals mindlessly completing surveys,^10,11^ or who did not complete the survey (n=29). Figure S1 provides a flow diagram showing sample construction. The survey remained in the field until 1,515 surveys that passed the attention check were completed, a number determined by the cost of a completed survey and available funding.

**Section 1.2: Internal Consistency of Survey Responses**

We checked whether responses were internally consistent to safeguard against further inattention and false responses. For our first test, we evaluated how people responded that they were personally impacted by opioids among respondents who self-reported no prescription opioid use in the past 12 months, no prescription opioid misuse in the past 12 months, and no use of non-prescription opioids in the past 12 months (N=1,107). Figure S2 reports this sample’s responses regarding how they have been personally impacted by opioids. Respondents could select more than one option for this question. 95.48% (95% CI: 94.26% - 96.71%) reported no impact while a small share (3.25% [95% CI: 2.21% - 4.30%]) reported that they struggled with dependence and 1.72% (95% CI: 0.95% - 2.48%) reported that they sought treatment. These latter answers would be consistent with opioid dependence issues over 12 months before the survey. The low rates of respondents in this sample claiming any prior impacts of opioids to their life is consistent with the absence of opioid use and misuse within the past 12 months.

Next, we evaluated the self-reported rates of overdose likelihood among respondents who self-reported no prescription opioid use in the past 12 months, no prescription opioid misuse in the past 12 months, no use of non-prescription opioids in the past 12 months, and reported that they have not been personally impacted by opioids (N=1,057). These selections do not imply that the respondent has never had exposure to opioids, nor does it imply that they might not anticipate an overdose involving opioids if, for example, they frequently use illicit substances and are aware that illicitly-made fentanyl could potentially contaminate those substances. Figure S3 shows that 92.90% (95% CI: 91.36% - 94.45%) of these respondents reported it was unlikely that they would overdose from opioid use. This high rate suggests that the responses were internally consistent.

**Section 1.3 Comparison to the May 2024 Current Population Survey (CPS)**

Our sample included 1,515 completed survey responses. The first questions in the survey asked for demographic information. We compared the demographics of our sample to the May 2024 Current Population Survey (CPS), the most recent monthly CPS available. The CPS is the primary source of monthly labor force statistics in the United States and provides detailed information on the demographic characteristics of US residents. Table S1 provides the demographic characteristics for the Respondi sample (column 1) and for the population aged 18 and over in the May 2024 CPS (column 2).

The Respondi sample is similar based on gender to the CPS, with about 49% in each sample identifying as male. We permitted respondents in our survey to select male or female or to self-identify. The CPS does not provide this latter option.

The survey also asked about the respondent’s race. Respondents could select one or more options regarding their race. Given the small sample size, we grouped respondents into “Black,” “White,” and “Other,” in which the last category includes all other responses and anyone who selected more than one race. The Respondi sample had higher shares of individuals identifying as Black or “Other Race” compared to the CPS and a lower share identifying as White.

The survey also asked about Hispanic ethnicity, and we found that the Respondi sample and the CPS had similar proportions of individuals identifying as Hispanic. The age distribution was also similar, although our sample had a smaller share of respondents aged 75+ and higher shares at younger parts of the age distribution. The largest difference between the Respondi sample and the CPS related to educational attainment. The Respondi sample was less likely to have never attended any college (i.e., high school degree or less). The geographical distribution of survey respondents, based on Census region, was similar to that of the CPS.

In summary, the survey sample looked similar to the US population aged 18 and over based on gender, race, Hispanic ethnicity and region of the country (Table S1). The sample differed most clearly based on education: the percentage of respondents with no college experience was low relative to the population, a difference that may overstate knowledge of OTC naloxone.

Figure S1. Flow Diagram of Sample Construction


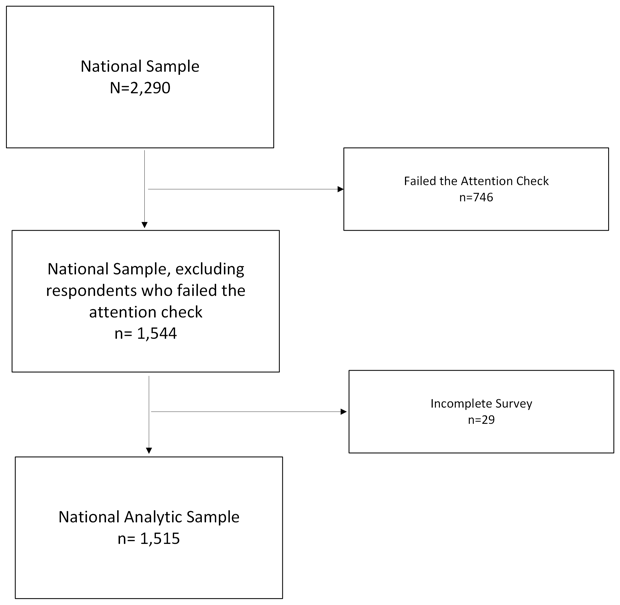


Figure S2: Personal Impacts of Opioid Use Among Respondents with No Prescription or Illicit Opioid Use within Past 12 Months (N=1,107)


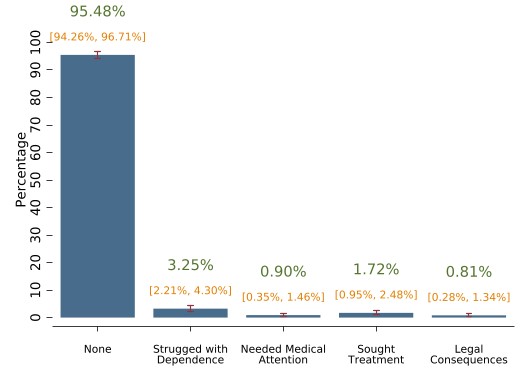


*Notes: Respondents could select “None” or at least 1 of the other options.*

Figure S3: Self-Reported Likelihood of Overdosing from Opioid Use for Respondents with No Prescription or Illicit Opioid Use within Past 12 Months and No Personal Impact from Opioid Use (N=1,057)


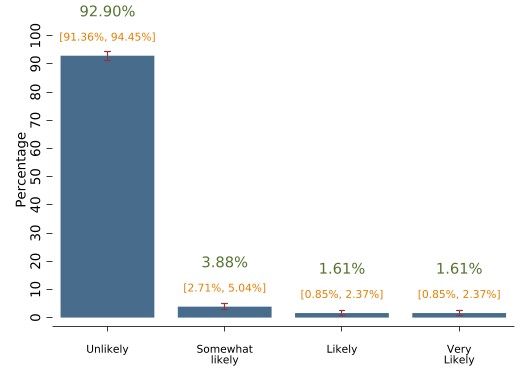


Table S1: Demographic and Geographic Characteristics of the Survey Sample with

the May 2024 Current Population Study

*Notes: The last column provides population shares using the May 2024 Current Population Study (CPS) and their population-weights. Educational attainment is summarized for the population ages 25+. Due to small sample sizes in our survey, we aggregated self-reported race into 3 categories.*

**References**

1. Alesina A, Miano A, Stantcheva S. Immigration and redistribution. *The Review of Economic Studies*. 2023;90(1):1-39.

2. Alesina A, Stantcheva S, Teso E. Intergenerational mobility and preferences for redistribution. *American Economic Review*. 2018;108(2):521-554.

3. Stantcheva S. Understanding tax policy: How do people reason? *The Quarterly Journal of Economics*. 2021;136(4):2309-2369.

4. Kerr JR, Schneider CR, Recchia G, et al. Correlates of intended COVID-19 vaccine acceptance across time and countries: results from a series of cross-sectional surveys. *BMJ open*. 2021;11(8):e048025.

5. Moscardino U, Musso P, Inguglia C, Ceccon C, Miconi D, Rousseau C. Sociodemographic and psychological correlates of COVID-19 vaccine hesitancy and resistance in the young adult population in Italy. *Vaccine*. 2022;40(16):2379-2387.

6. Roth AE, Wang SW. Popular repugnance contrasts with legal bans on controversial markets. *Proceedings of the National Academy of Sciences*. 2020;117(33):19792-19798.

7. Liscow Z, Pershing A. Why is so much redistribution in-kind and not in cash? Evidence from a survey experiment. *National Tax Journal*. 2022;75(2):313-354.

8. Schyns B, Kiefer T, Foti RJ. Does thinking of myself as leader make me want to lead? The role of congruence in self-theories and implicit leadership theories in motivation to lead. *Journal of Vocational Behavior*. 2020;122:103477.

9. Wegwarth O, Mansmann U, Zepp F, Lühmann D, Hertwig R, Scherer M. Vaccination intention following receipt of vaccine information through interactive simulation vs text among COVID-19 vaccine–hesitant adults during the omicron wave in Germany. *JAMA Network Open*. 2023;6(2):e2256208-e2256208.

10. Oppenheimer DM, Meyvis T, Davidenko N. Instructional manipulation checks: Detecting satisficing to increase statistical power. *Journal of Experimental Social Psychology*. 2009/07/01/ 2009;45(4):867-872. doi:<https://doi.org/10.1016/j.jesp.2009.03.009>

11. Paas LJ, Morren M. Please do not answer if you are reading this: Respondent attention in online panels. *Marketing Letters: A Journal of Research in Marketing*. 2018;29(1):13-21. doi:10.1007/s11002-018-9448-7
